# Ultra-Narrowband Anisotropic Perfect Absorber Based on α-MoO_3_ Metamaterials in the Visible Light Region

**DOI:** 10.3390/nano12081375

**Published:** 2022-04-17

**Authors:** Gui Jin, Tianle Zhou, Bin Tang

**Affiliations:** 1 Department of Electronic Information and Electronic Engineering, Xiangnan University, Chenzhou 423000, China; jingui0531@exu.edu.cn; 2 School of Microelectronics and Control Engineering, Changzhou University, Changzhou 213163, China; 00001900@cczu.edu.cn

**Keywords:** perfect absorber, α-phase molybdenum trioxide (α-MoO_3_), metamaterials, polarization

## Abstract

Optically anisotropic materials show important advantages in constructing polarization-dependent optical devices. Very recently, a new type of two-dimensional van der Waals (vdW) material, known as α-phase molybdenum trioxide (α-MoO_3_), has sparked considerable interest owing to its highly anisotropic characteristics. In this work, we theoretically present an anisotropic metamaterial absorber composed of α-MoO_3_ rings and dielectric layer stacking on a metallic mirror. The designed absorber can exhibit ultra-narrowband perfect absorption for polarizations along [100] and [001] crystalline directions in the visible light region. Plus, the influences of some geometric parameters on the optical absorption spectra are discussed. Meanwhile, the proposed ultra-narrowband anisotropic perfect absorber has an excellent angular tolerance for the case of oblique incidence. Interestingly, the single-band perfect absorption in our proposed metamaterials can be arbitrarily extended to multi-band perfect absorption by adjusting the thickness of dielectric layer. The physical mechanism can be explained by the interference theory in Fabry–Pérot cavity, which is consistent with the numerical simulation. Our research results have some potential applications in designs of anisotropic optical devices with tunable spectrum and selective polarization in the visible light region.

## 1. Introduction

Two-dimensional (2D) materials with atomic-scale thicknesses, i.e., graphene [1], black phosphorus (BP) [2], hexagonal boron nitride (h-BN) [3], and transition metal dichalcogenides (TMDs) [4], have been much concerned due to their distinctive optical and electrical properties over the past few years [5]. Different from the optoelectronic devices made of conventional bulk materials, these layered materials may provide exciting opportunities for designing novel optoelectronic applications. Very recently, a new type of 2D van der Waals (vdW) material, known as α-phase molybdenum trioxide (α-MoO_3_), has been experimentally demonstrated and sparked considerable interest due to its highly anisotropic characteristics stemming from the unique crystalline structure [6,7,8]. In fact, most of the van der Waals materials, i.e., molybdenum disulphide (MoS_2_), in which the central Mo atom in MoS_2_ is sandwiched between two Sulphur atoms, are uniaxial crystals. However, α-MoO_3_ is actually a type of natural biaxial hyperbolic crystal, and it exhibits pristine in-plane hyperbolic dispersion. The structure of α-MoO_3_ is constructed by layers of distorted octahedral crystal [9], in which Mo atoms are separately linked with three different oxygen atoms, i.e., symmetrically bridging O_s_, terminal O_t_, and asymmetric O_a_. Each α-MoO_3_ layer consists of two sub-layers, which are created by corner-sharing rows and edge-sharing zigzag rows. Therefore, α-MoO_3_ crystal materials can be combined into metamaterials to achieve more degree of freedom for manipulating light-matter interaction at nanoscale. Moreover, the strong anisotropy of α-MoO_3_ materials could be useful for a wealth of applications ranging from color filter [10], polarization converter [11], and molecular sensors to in-plane imaging [12]. Besides, some other exotic physical phenomena were observed by exploiting phonon polaritons excited in α-MoO_3_. For instance, Qu et al. reported a tunable planar focusing nanophotonic device working in the mid-infrared region [13]. Hu et al. experimentally explored the topological transitions and photonic magic angle in twisted bilayered α-MoO_3_ flakes [14].

Metamaterials, known as artificial composite materials composed of periodical sub-wavelength scale nanostructure, possess some exotic electromagnetic characteristics that are not found in natural materials [15,16]. To date, tremendous interest has been attracted for their extensive applications [17,18]. As an important branch of metamaterials, metamaterial absorber manifests intriguing strategies for its relatively flexible design in comparison with the conventional electromagnetic absorber based on bulky components. Since the first experimental demonstration [19], many types of metamaterial absorbers with narrow-band absorption [20,21], broadband absorption [22,23], and even multi-band absorption [24,25], have been proposed owing to their wide applications, such as solar cells [26], plasmonic sensors [27], molecular detectors [28], and selective thermal emitters [29]. By reasonably designing the geometric structure, the operating frequencies of metamaterial absorbers can be run from microwaves to optical spectral regime. So far, various of 2D materials-based metamaterials have also been proposed for obtaining tunable perfect absorption or optical enhancement absorbance [30,31,32,33,34,35,36,37]. For example, Thongrattanasiri et al. demonstrated the complete optical absorption in periodically patterned graphene sheet [38]. Sang et al. proposed a two-band absorber utilizing the patterned MoS_2_ [39]. Zhu et al. presented tunable wide-angle and ultra-broadband perfect absorbers by using BP-dielectric multi-layer stacking structure and BP-dielectric-metallic hybrid architecture [40,41], respectively. However, to our knowledge, the understanding of the interaction of light with α-MoO_3_ materials is still in its infancy, and few works have been reported on the electromagnetic absorbers based on α-MoO_3_ [42,43,44], especially for ultra-narrowband anisotropic perfect absorption, which are of significance for some applications, such as photodetectors, spectral imaging, and sensors.

In this work, we theoretically propose and numerically demonstrate an ultra-narrowband anisotropic metamaterial absorber composed by top α-MoO_3_ ring and a dielectric layer stacked on metallic mirror. By the appropriate design of this structure, ultra-narrowband perfect absorption can be achieved in visible frequency for polarization along both *x*- and *y*-directions. Meanwhile, the proposed anisotropic metamaterial absorber has an excellent angular tolerance for the case of oblique incidence. More interestingly, the single-band perfect absorption in our proposed metamaterials can be arbitrarily extended to multi-band perfect absorption by adjusting the thickness of dielectric layer. The physical mechanism can be explained by the interference theory in Fabry–Pérot cavity, which is consistent with the numerical simulation. Moreover, the electromagnetic simulations performed by finite-difference time-domain (FDTD) method match well with the results of theoretical analysis. Our investigation shows promising potential in sensing, multispectral detection, filters and multiplexing binding bio-molecular detection, etc.

## 2. Structural Design and Simulation

**Figure 1:**
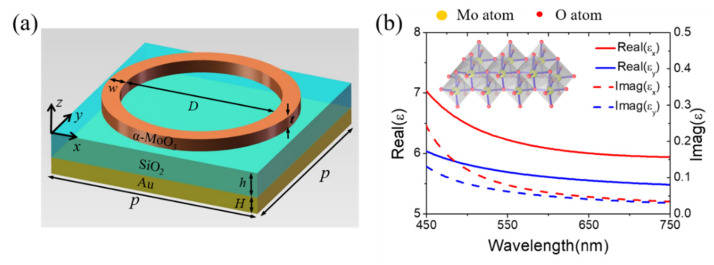
(**a**) The structure unit cell diagram of the proposed absorber consisting of top α-MoO_3_ ring and SiO_2_ layer stacked on a gold substrate. In this design, the geometrical parameters are listed as follows: *H* = 200 nm, *h* = 300 nm, *w* = 40 nm, *D* = 360 nm, *p* = 500 nm, and the thickness of α-MoO_3_ ring *t* = 105 nm. (**b**) The real part and imaginary part of the α-MoO_3_ permittivity in the visible region. The inset is the schematic of the α-MoO_3_ material with layered structure. The yellow and red spheres represent molybdenum and oxygen atoms, respectively.

Figure 1a shows schematically the unit cell of proposed anisotropic perfect absorber, which consists of top α-MoO_3_ ring and dielectric layer stacked on a gold mirror. The relevant geometric parameters and values are listed in the caption of Figure 1. As far as the process of fabrication, the multilayer structure can be fabricated by using physical vapor deposition techniques, which has been a well-known method for scalable and repeatable synthesis. In our design, the complex dielectric function of α-MoO_3_ can be described as follows [10]:

(1) $${\varepsilon (\omega )=\varepsilon }_{\infty }+\underset{i}{\sum }\frac{\omega_{\mathrm{pi}}^{2}}{\omega_{\mathrm{oi}}^{2}{+\omega }^{2}{-i\gamma }_{i}\omega }$$

where *i* indicates the number of Lorentz oscillators, *ε*_∞_, *ω_pi_*, *ω_oi_*, and *γ_i_* refers to the high frequency dielectric constant, the plasma frequency, the eigenfrequency, and the scattering rate of the *i*th Lorentz oscillator, respectively. The parameters used in Equation (1) to calculate the permittivity tensors of α-MoO_3_ are listed in Table 1 [10].

Figure 1b illustrates the real and imaginary parts of α-MoO_3_ permittivity along [100] and [001] directions extracted from Ref. [10]. Full field electromagnetic calculations were performed by using Lumerical FDTD Solution software package. The three-dimensional FDTD simulations were made in a unit cell area, and the non-uniform mesh is chosen, and the mesh size gradually increases outside the α-MoO_3_ material. Following the crystallographic direction conventions, the *x*-, *y*-, and *z*-directions represent the [100], [001], and [010] directions, respectively. In calculations, the plane waves were illuminated along the negative *z*-direction, and periodic boundary conditions were used in *x*- and *y*-directions. In general, the optical absorption can be expressed as *A* = 1 − *R* − *T*, where *R* and *T* indicate the reflection and transmission, respectively. Considering that the thickness of the gold mirror has exceeded the skin depth of the light, the transmission *T* is equal to zero. Therefore, the absorption coefficient *A* can be abbreviated as *A* = 1 − *R*. In addition, the dielectric layer is chosen to be SiO_2_ with a refractive index of 1.45, and the permittivity of gold is described by Drude model:

(2) $$\varepsilon \left(\omega \right)=\varepsilon_{\infty }-\frac{\omega_{p}^{2}}{\omega^{2}+i\omega \gamma }$$

where *ω* is the angular frequency, and the plasma frequency *ω_p_* is 1.37 × 10^16^ rad/s, the scattering rate *γ* = 4.08 × 10^13^ rad/s, and *ε_∞_* = 1.

## 3. Results and Discussion

**Figure 2:**
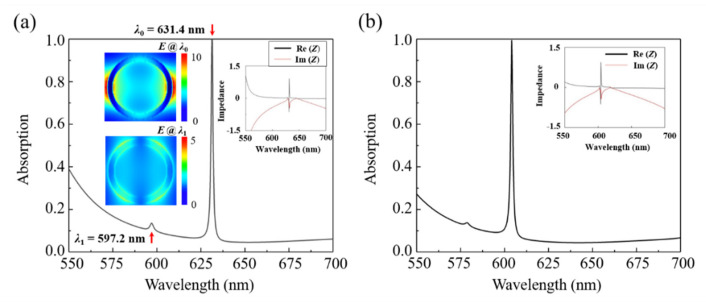
(**a**) Simulated absorption spectra for the proposed α-MoO_3_ absorber structure along the [100] directions. (**b**) Simulated absorption spectra for the proposed α-MoO_3_ absorber structure along the [001] directions. The subgraphs of Figure 2 represent the calculated real and imaginary parts of the effective impedance along the [100] and [001] directions, respectively.

Figure 2 gives the optical absorption spectra of the designed metamaterial absorber when the polarization directions of incident lights are along the [100] and [001] crystal direction of α-MoO_3_, respectively. The simulation results show that the proposed absorber can achieve perfect absorption in both [100] and [001] crystal directions. It is shown from Figure 2a that the designed nanostructure can achieve an absorptivity of 99.72% at the wavelength of 631.4 nm when the polarization direction of light is along [100] crystal direction of α-MoO_3_. Meanwhile, there exists a weak absorption peak at 597.2 nm with absorptivity of 16.2%. In contrast, when the polarization direction of light is along [001] crystal direction of α-MoO_3_, the absorptivity reaches up to 99.28% at the resonant wavelength of 604.1 nm as shown in Figure 2b. The shift of resonance wavelength is attributable to the strong anisotropy of α-MoO_3_. Besides, according to the definition of quality factor *Q*, the total *Q* factor can be written by *Q* = *f*_0_/Δ*f,* in which *f*_0_ corresponds to the resonance frequency at the peak wavelength, and Δ*f* represents the full width at half maximum. Thus, the calculated values of quality factors amount to 538.30 and 574.95 in the proposed α-MoO_3_–based metamaterial absorber for polarizations along [100] and [001] crystalline directions, respectively. In addition, the effective impedances of the absorber in the visible region are calculated as shown in the insets of Figure 2a,b. It is known that under critical coupling conditions, the effective impedance of structure system matches with that of free space (*Z* = *Z*_0_ = 1), which can be expressed as in which $Z=\sqrt{\frac{{\left({1+S}_{11}\right)}^{2}{\;-\;S}_{21}^{2}}{{\left({1–S}_{21}\right)}^{2}{\;-\;S}_{21}^{2}}}$, where S_11_ and S_21_ denote as the scattering parameters relevant to reflectance and transmittance coefficient [45]. When the polarization directions are along the [100] and [001] crystal directions of α-MoO_3_, the corresponding effective impedances are *Z*_1_ = 0.92 − 0.12*i* and *Z*_2_ = 0.94 − 0.10*i*, respectively. It is demonstrated that the effective impedance of the absorber system matches well with the normalized impedance of the free space, which effectively suppresses the light reflection and achieves the perfect absorption. In order to manifest the underlying physical mechanism behind the resonant absorption phenomenon, the field distributions at the resonant wavelengths are given in the inset for polarization along [100] direction of α-MoO_3_. As shown in the insets, the electric field distribution at the resonance wavelength of 631.38 nm in *x*-*y* plane shows the characteristics of electric dipole, which is symmetric about the *y*-axis in viewing of the incidence polarization along [100] direction of α-MoO_3_. The localized collective electron excitations strongly couple with the incidence light, thus leading to the perfect electromagnetic waves absorption. Meanwhile, one can observe that there exists a weak resonance absorption peak at the wavelength of 597.2 nm, which is attributable to the excitation of electric quadrupole around the edge of α-MoO_3_ ring. Similarly, the operation mechanism also applies for the case of polarization along [001] crystal directions of α-MoO_3_.

**Figure 3:**
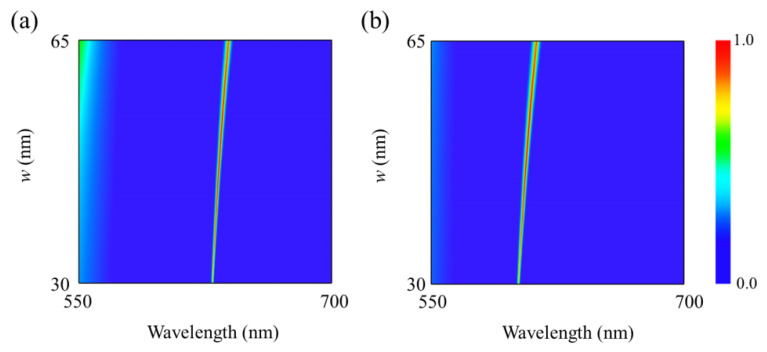
Absorption spectra of the proposed structure as a function of wavelength and width *w* of nanoring for polarizations along (**a**) [100] and (**b**) [001] crystalline directions of α-MoO_3_. *h* = 300 nm, *p* = 500 nm, and *t* = 105 nm.

**Figure 4:**
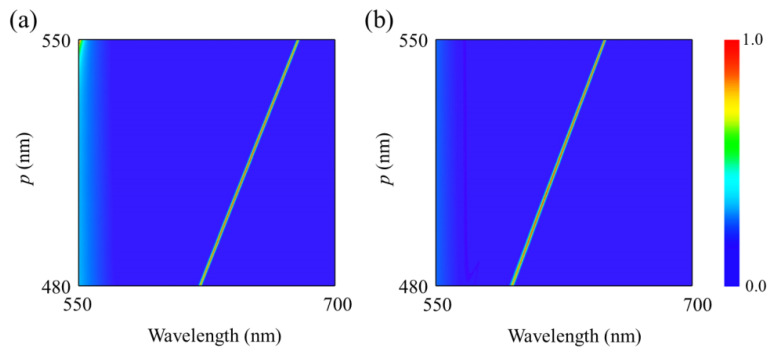
Absorption spectra of the proposed structure as a function of wavelength and period *p* for polarizations along (**a**) [100] and (**b**) [001] crystalline directions of α-MoO_3_. *h* = 300 nm, *w* = 40 nm, and *t* = 105 nm.

To study the influences of geometric parameters on the optical absorption spectra, Figure 3 and Figure 4 calculate the optical absorption spectra as a function of wavelength and the width *w* of α-MoO_3_ nanoring as well as the period *p* of nanostructure. One can see from Figure 3 that when the width of α-MoO_3_ nanoring is increasing from 30 to 65 nm, the resonant wavelength of the absorptive spectra has a slight redshift for polarization along the [100] and [001] directions, respectively. Meanwhile, the absorption efficiency has an obvious enhancement with increasing of *w*. From Figure 4, it is clearly shown that the absorption spectra are closely related to the periodicity of nanostructure array. When the period *p* is increased from 480 to 550 nm, the resonant absorption peaks shift to the longer wavelength, and the different resonant wavelength for both polarizations originates from the strong anisotropy of lattice structure of α-MoO_3_ crystal. Therefore, one can modulate the optical absorption spectra by controlling the related geometric parameters.

**Figure 5:**
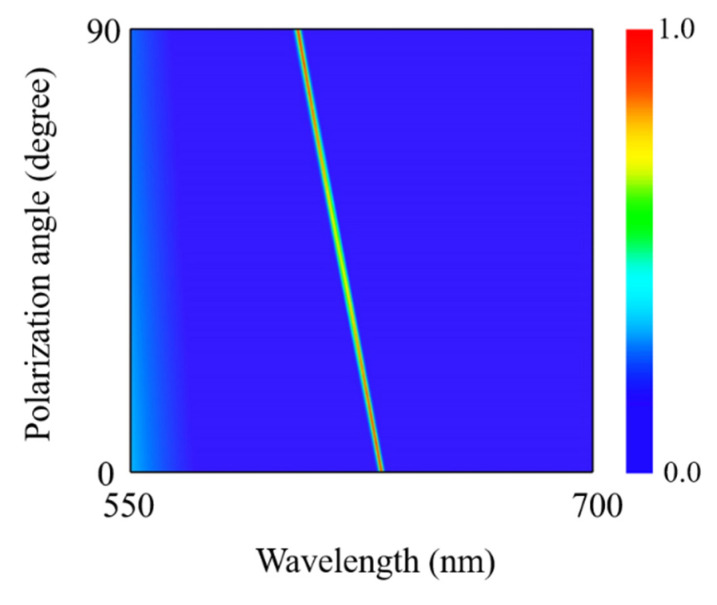
Absorption spectra of the proposed structure as a function of wavelength and polarization angle of incident light. *P* = 500 nm, *w* = 40 nm, *t* = 105 nm, and *h* = 300 nm.

**Figure 6:**
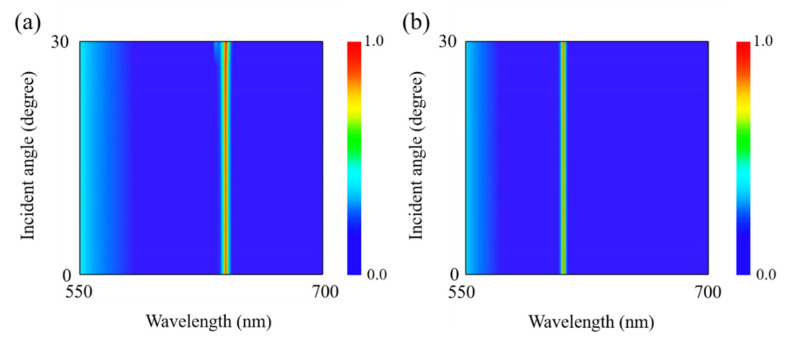
Absorption spectra of the proposed structure as a function of wavelength and the incident angle with polarization along (**a**) [100] and (**b**) [001] crystalline directions of α-MoO_3_. *p* = 500 nm, *w* = 40 nm, *t* = 105 nm, and *h* = 300 nm.

To further explore the influences of polarization direction on the optical absorption spectra, Figure 5 calculates the optical absorption spectra as a function of wavelength and polarization angle. One can see from Figure 5 that when the polarization angle is gradually increased from 0° (*x*-polarization) to 90° (*y*-polarization), the optical spectrum displays an ultra-narrowband resonant absorption peak, which has also a blue-shift from 631.4 nm to 604.1 nm due to the anisotropic lattice structure of α-MoO_3_. Meanwhile, the absorptivity at the resonant wavelength also changes with increasing of the polarization angle. Especially, the optical absorptivity reaches up to perfect absorption when the polarization angle takes the value of 0° or 90°. Therefore, the optical absorption spectra exhibit a polarization-dependent characteristic even though the geometric structure is circularly symmetric. Furthermore, the absorption performance of the absorber under oblique incidence is also investigated. Figure 6 calculates the optical absorption spectra as a function of wavelength and incident angle. It can be clearly seen from Figure 6 that when the incident angle is increased from 0° to 30°, the optical spectra take on an ultra-narrowband perfect absorption for both polarizations along [100] and [001] directions of α-MoO_3_, respectively. The absorption performance in both [100] and [001] directions is insensitive to the incident angle. Meanwhile, the positions of resonant absorption peaks almost keep invariant for both polarization directions. Comparing Figure 6a,b, the resonant wavelength has a blue shift when the polarization is switched from [100] direction to [001] direction of α-MoO_3_.

**Figure 7:**
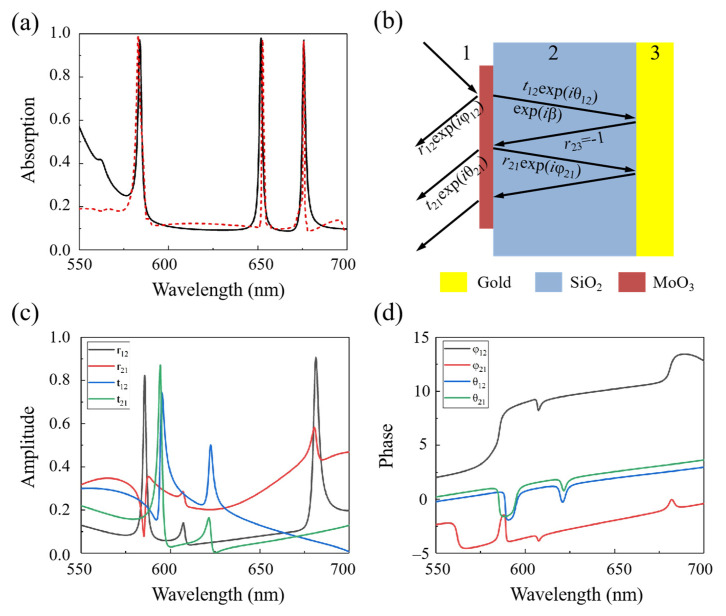
(**a**) The simulated absorption spectrum (black curve) of the absorber with a thickness of *h* = 500 nm and the calculated absorption spectrum based on the interference theory (red curve). (**b**) The interference theory model of the propose absorber. (**c**) Amplitude and (**d**) phase of the reflection and transmission coefficients obtained from proposed absorber, respectively.

The above mentioned single-band perfect absorption can be extended to multi-band absorption as shown in Figure 7a. It can be seen from Figure 7a that there are three nearly perfect absorption bands when the dielectric spacer takes the value of 500 nm as shown by the black line. The phenomenon of triple-band absorption can be explained by interference theory in Fabry–Pérot cavity. As shown in the Figure 7b, the incident light is partially reflected back to air with a reflection coefficient $\overset{\sim }{r}$_12_ = *r*_12_*e^iφ^*^12^ and partially transmitted into the dielectric layer with a transmission coefficient $\overset{\sim }{t}$_12_ = *t*_12_*e^i^^θ^*^12^ at the air-spacer interface with α-MoO_3_ ring. In the model of interference theory, the lights are reflected back and forth between the α-MoO_3_ ring and metal substrate, with a complex propagation phase β = $\sqrt{\overset{\sim }{\varepsilon }}k_{0}h$, where $\overset{\sim }{\varepsilon }$ is the permittivity of the dielectric spacer, *k*_0_ is the wavenumber of free space, and *h* is the thickness of the dielectric spacer. Plus, there appear partial reflection with coefficients $\overset{\sim }{r}$_21_ = *r*_21_*e^iφ^*^21^ and transmission with coefficients $\overset{\sim }{t}$_21_ = *t*_21_*e^i^^θ^*^21^ at the air-spacer interface with α-MoO_3_ ring. The total reflection is the superposition of the multiple reflection [46]:

(3) $$\overset{\sim }{r}={\overset{\sim }{r}}_{12}-\frac{{\overset{\sim }{t}}_{12}{\overset{\sim }{t}}_{21}e^{i2\beta }}{1+{\overset{\sim }{r}}_{21}e^{i2\beta }}$$

The absorbance can be calculated through $\overset{\sim }{A}$(*ω*) = 1 − |$\overset{\sim }{r}$(*ω*)|^2^, where $\overset{\sim }{r}$(*ω*) is the total reflection originating from the superposition of multiple reflection. Take the *h* = 500 nm of the proposed absorber as an example, as shown in the Figure 7a, the results of interference theory (red dashed line) are consistent well with the numerical simulations (black line). The amplitude and phase corresponding to $\overset{\sim }{t}$_12_, $\overset{\sim }{t}$_21_, $\overset{\sim }{r}$_12_, and $\overset{\sim }{r}$_21_ are shown in Figure 7c,d, respectively. Therefore, one can conclude that the proposed absorber can obtain arbitrary number of absorption bands in the visible light region by choosing appropriate thickness of the dielectric layer.

**Figure 8:**
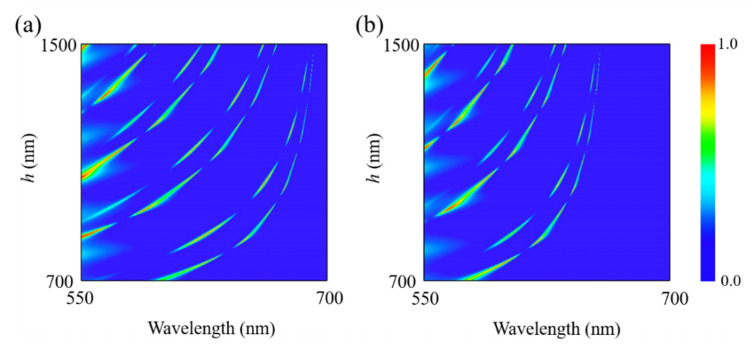
Absorption spectra of the proposed structure as a function of wavelength and the thickness of dielectric layer *h* for incident polarization along (**a**) [100] and (**b**) [001] crystalline directions of α-MoO_3_. *p* = 500 nm, *w* = 40 nm, and *t* = 105 nm.

To demonstrate the effect of dielectric layer thickness *h* on the resonant absorption of the structure, Figure 8 gives the absorption spectra of the proposed nanostructure as a function of wavelength and the dielectric layer thickness. It can be clearly seen from Figure 8 that for polarizations along the [100] and [001] crystal directions of α-MoO_3_, the resonance absorption of the absorber can be adjusted from one peak to multiple peaks by choosing suitable dielectric layer thickness, i.e., *h* = 1500 nm. Similar to the triple-band absorption phenomena, the multi-band absorption can also be interpreted by interference theory in Fabry–Pérot cavity. Plus, one can conclude from the model of interference theory that the dielectric thickness plays an important role in determining the resonance wavelength of Fabry–Pérot cavity. As a result, with increasing of the dielectric layer thickness, all the resonance peaks exhibit the tendency of redshift. The maximum optical absorbance appears at the constructive interference with approximate phase match condition of 2β+φ+π≈2mπ, where *φ* means the phase shift, and *m* is an integer.

## 4. Conclusions

In conclusion, we theoretically proposed and numerically demonstrated an ultra-narrowband anisotropic metamaterial perfect absorber based on α-MoO_3_, unit cell of which consists of an α-MoO_3_ ring and dielectric layer stacked on a gold mirror. The numerical results show that the ultra-narrowband perfect absorption can be obtained in the visible light band for polarizations along the [100] and [001] crystal directions of α-MoO_3_. Plus, the influences of some geometric parameters on the optical absorption spectra are discussed. Meanwhile, the proposed anisotropic metamaterial absorber has an excellent angular tolerance for the case of oblique incidence. Especially, the single-band perfect absorption in our proposed metamaterials can be arbitrarily developed into multi-band perfect absorption by choosing the suitable thickness of dielectric layer. The physical mechanism can be explained by the interference theory in Fabry–Pérot cavity, which is consistent with the numerical simulation. Our research results have some potential applications in designs of anisotropic meta-devices with tunable spectra and selective polarization in the visible light region.

## Figures and Tables

**Table 1:** Parameters used in Equation (1) to obtain the permittivity tensors of α-MoO_3_ in the visible range.

Polarization	*ε*_∞_	*ω_pj_* [cm^−1^]	*ω*_0_ [cm^−1^]	*γ_j_* [cm^−1^]
*x*	5.065	21,672	27,019	1342.2
*y*	4.502	29,078	32,271	2027.1

## Data Availability

The data are included in the main text.
